# An NFκB Activity Calculator to Delineate Signaling Crosstalk: Type I and II Interferons Enhance NFκB via Distinct Mechanisms

**DOI:** 10.3389/fimmu.2019.01425

**Published:** 2019-06-25

**Authors:** Simon Mitchell, Ellen L. Mercado, Adewunmi Adelaja, Jessica Q. Ho, Quen J. Cheng, Gourisankar Ghosh, Alexander Hoffmann

**Affiliations:** ^1^Signaling Systems Laboratory, Institute for Quantitative and Computational Biosciences, Department of Microbiology, Immunology, and Molecular Genetics, and Molecular Biology Institute, University of California Los Angeles, Los Angeles, CA, United States; ^2^Signaling Systems Laboratory, San Diego Center for Systems Biology, La Jolla, CA, United States; ^3^Department of Chemistry and Biochemistry, University of California, San Diego, La Jolla, CA, United States

**Keywords:** mathematical model, signaling crosstalk, interferon, NFκB, systems biology, translational inhibition, immunoproteasome, anti-viral response

## Abstract

Nuclear factor kappa B (NFκB) is a transcription factor that controls inflammation and cell survival. In clinical histology, elevated NFκB activity is a hallmark of poor prognosis in inflammatory disease and cancer, and may be the result of a combination of diverse micro-environmental constituents. While previous quantitative studies of NFκB focused on its signaling dynamics in single cells, we address here how multiple stimuli may combine to control tissue level NFκB activity. We present a novel, simplified model of NFκB (SiMoN) that functions as an NFκB activity calculator. We demonstrate its utility by exploring how type I and type II interferons modulate NFκB activity in macrophages. Whereas, type I IFNs potentiate NFκB activity by inhibiting translation of IκBα and by elevating viral RNA sensor (RIG-I) expression, type II IFN amplifies NFκB activity by increasing the degradation of free IκB through transcriptional induction of proteasomal cap components (PA28). Both cross-regulatory mechanisms amplify NFκB activation in response to weaker (viral) inducers, while responses to stronger (bacterial or cytokine) inducers remain largely unaffected. Our work demonstrates how the NFκB calculator can reveal distinct mechanisms of crosstalk on NFκB activity in interferon-containing microenvironments.

## Introduction

NFκB is the primary transcriptional regulator of inflammation (1), controlling the expression of inflammatory cytokines and chemokines that activate and coordinate both local and systemic immune responses, as well as tissue remodeling factors that facilitate immune cell invasion and tissue repair (2). Furthermore, NFκB controls cell survival genes and its activity is associated with chemoresistance in cancer cells (3). As a result, high NFκB activity in chronic disease is often associated with poor prognosis (4). Indeed, clinical histological screening to inform treatment strategies often involves assessment of NFκB expression or activity (5, 6).

The molecular mechanisms by which the primary NFκB protein RelA is activated in response to inflammatory cytokines or pathogen exposure have been elucidated. Inflammatory stimuli induce phosphorylation by the IκB kinase (IKK) complex of IκBs, triggering their ubiquitin-dependent proteasomal degradation, and thus freeing NFκB to translocate to the nucleus to bind to DNA κB elements and induce transcription of target genes. NFκB target genes include several IκBs, which upon induction provide negative feedback on the system thus regulating the dynamics of NFκB activity (7–9).

Mathematical kinetic models of the IκB-NFκB signaling module have contributed to our understanding of the complex and often oscillatory dynamics of NFκB activity observed in single cells stimulated with a defined inflammatory agonist (10). However, due to cellular heterogeneity such oscillatory responses are rarely observed at the cell population level (11, 12). In primary or tissues cells such dynamic heterogeneity is likely to be even greater given their differential steady states (13). Indeed, in various clinical settings, overall NFκB activity in cell populations (average nuclear localization across many cells) examined in tissues has prognostic value. While recent studies have distributed the state of single-cell simulations to estimate cell population behavior (13, 14) such approaches are computationally challenging due to the need to numerically solve a large system of equations for each cell in the simulation. This may preclude comprehensive parameter scanning, preventing full characterization of possible responses. Only small models can be analytically solved to obtain concentrations without the need for relatively slow computational numerical solvers. In addition, due to the number of molecular species in larger models that cannot be experimentally measured, the iterative interpretation of experimental results with computational simulation can be challenging. For a given experimental observation, multiple reaction rates can often be perturbed to explain the result leading to challenges in targeting the next experiment. This calls for a simplified modeling framework that coarse-grains the known regulatory mechanisms when the data of interest do not demand detailed models. Simplified models of NFκB have previously been constructed and shown to be useful in elucidating the regulatory principles underlying its oscillatory control of single cells (15–18). However, no models have been reported that focus on the regulatory principles governing the quantitative average NFκB activity of many cells i.e., models that recapitulate the tissue scale NFκB activity. Though models representing the aggregate behavior of multiple cells or entire organs, i.e., tissue-scale models, are further abstractions of the regulatory mechanisms than models that recapitulate the intra-cellular regulatory dynamic, they have proven useful to investigate the dose-response and time-evolution of diverse biological phenomena, such as hormone control and the interplay between organ function, drug metabolism, and the responses to drugs (19–21).

One diverse cytokine family that defines tissue microenvironments are the interferons (22); the most prominent family members, IFNβ and IFNγ, exemplify type I and type II interferons, respectively. Interferons are typically coordinately activated with NFκB in sites of infection and play roles in inflammatory disease even if their primary physiological function is anti-viral gene expression. Indeed, both clinical and experimental studies point to crosstalk by interferons on NFκB-driven inflammatory signaling (23–27). For example, inflammatory symptoms and cytokine secretion during an infection with *streptococcus pneumoniae* are exacerbated by infection with influenza. Similar clinical symptoms during leishmaniasis are observed when the parasites harbor the Leishmania RNA Virus (LRV) (28, 29).

Laboratory studies have proposed two broad classes of cross-regulatory mechanisms: one mediated by chromatin, altering how induced NFκB controls gene expression, and the other mediated by the signaling networks, affecting the level of NFκB activity. In line with the former, IFN-mediated RNA pol II recruitment or IFN-mediated chromatin remodeling of NFκB-inducible genes have been identified as mechanisms potentiating inflammatory gene expression (30–34). In regards to the latter, IFNs have been reported to affect NFκB activity by altering signal transduction between TLRs and NFκB via expression of receptors, co-receptors and adapter proteins (35–41), or by altering translation control through phosphorylating eukaryotic initiation factors (eIF)2α and eIF4E, which may also diminish translation of IκBα (40, 42–45). However, these mechanisms must allow for a level of stimulus-specificity, as TLR4-mediated NFκB activation was, for example, found to be unaffected by IFNγ (34).

Here we construct a simple model of NFκB control, termed SiMoN, to capture the activity of populations of cells and employ it in an iterative and quantitative systems biology study to investigate how signaling crosstalk by micro-environmental type I and II IFNs influences NFκB signaling. We identify distinct, IFN type-specific mechanisms that amplify NFκB activation in a stimulus-specific manner.

## Results

### A Simplified Model of NFκB Activity for Studying Cross-Regulation

**Figure 1:**
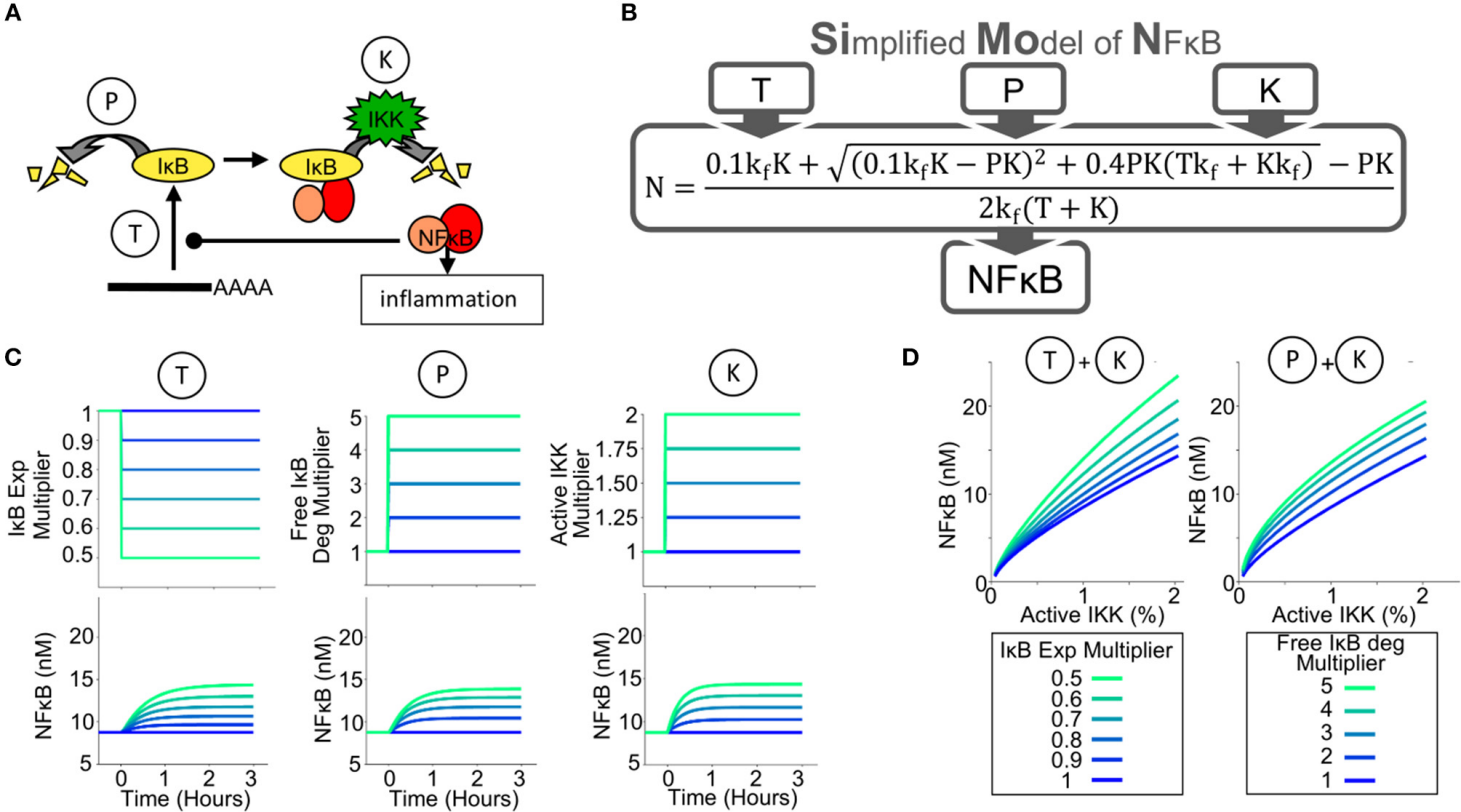
A Simplified Model of NFκB Activity (SiMoN) can predict NFκB activity from 3 parameters. **(A)** Schematic of the key reactions controlling NFκB activity through IκB metabolism. The amount of free, transcriptionally active, NFκB (NFκB activity) is tightly controlled by the amount of IκB; therefore IκB synthesis (reaction T) and free IκB degradation (reaction P) may potentially offer alternative points of control. The primary, canonical activation pathway is through IKK (reaction K), however, interferons do not directly activate IKK. **(B)** Schematic of the Simplified Model of NFκB (SiMoN), which analytically calculates NFκB as a result of parameters T,P and K. **(C)** Modeled time-course concentrations of free NFκB (lower), in response to perturbed reaction rates obtained by multiplying the WT parameter value by the multiplier indicated (upper) utilizing the simplified model. **(D)** Steady-state free NFκB concentrations in response to: increased IKK activity and IκB translation inhibition (left) and increased IKK activity and free IκB degradation (right).

Previously published mathematical models accurately recapitulate transient NFκB activities and oscillations caused by stimuli such as TNF or LPS (11, 12, 46–48) in fibroblasts and a macrophage cell line (49); these studies focused on a single enzymatic reaction that controls NFκB-activation: the IKK-mediated degradation of NFκB-bound IκB. To investigate the tissue scale control of NFκB and assist our intuitive understanding, a new mathematical model was constructed. To develop this simple quantitative tool we carefully considered the enzymatic reactions that control NFκB activity. Conceptualizing an abstracted model, we find that the amount of NFκB that is capable of binding DNA in the nucleus is determined by the abundance of the inhibitory IκB proteins, which in turn is a function of the biochemical reactions governing IκB synthesis and degradation (50). NFκB-bound IκBα is degraded through an IKK-mediated pathway, but free IκBα, that is IκBα not bound to NFκB, has a short half-life (51) determined by an IKK- and ubiquitination-independent pathway (Figure 1A). Thus, in principle, IKK-mediated NFκB activity (reaction K, Figure 1A) may be enhanced by reductions in IκB protein synthesis (reaction T, Figure 1A) or in the free IκB half-life (reaction P, Figure 1A).

The schema of the Simplified Model of NFκB (SiMoN) is given in Supplementary Figure 1 in Systems Biology Graphical Notation (52) and consists of three ordinary differential equations (ODEs) representing the rate of change of free (active) NFκB, free IκB, and the NFκB-IκB complex. The concentration of each constituent is a function of IκB synthesis, free IκB degradation (an IKK-independent process) and degradation of IκB from the IκB-NFκB complex (an IKK-dependent process) (Figure 1B, parameters T, P, and K, respectively). SiMoN approximates the average of multiple single cell simulations of TLR NFκB responses (Supplementary Figure 2). Although this model lacks the complexity of other NFκB signaling models that describe the highly dynamic and variable NFκB responses at single cell resolution (10), it provides for a simplified, intuitive understanding of the reactions that may be perturbed by signaling crosstalk and carry physiological relevance within populations of cells. In addition to these benefits of interpretation, SiMoN provides analytical benefits over single-cell models. Indeed, by assuming that the network reaches a steady-state quickly when reaction rates change (the quasi-steady-state assumption), SiMoN can avoid the need for simulation with numerical differential equation solvers. An analytical solution for the quasi-steady-state concentration of NFκB as a function of the kinase activity of IKK (K), free IκB protein degradation (P), and IκB synthesis via translation (T) was found (Figure 1B). NFκB activity can thus be directly calculated when the values of these parameters are known, and experimentally-measured changes in these parameters can be directly interpreted.

We used SiMoN to examine how NFκB activity is a function, not only of IKK activity, but also of translation inhibition and IKK-independent free IκB degradation. Steady-state concentrations of free NFκB were calculated to be increased by either increasing active IKK, inhibiting IκB translation or increasing free IκB degradation (Figure 1C). Dose response analyses suggest that both inhibition of IκB synthesis and free IκB degradation substantially amplify the response of free NFκB to increasing IKK activity (Figure 1D). This means that environmental conditions that do not activate IKK or alter its activity may nevertheless potentiate or modulate NFκB activity. To establish whether analytically investigating NFκB with SiMoN could elucidate mechanisms of cross-regulation we turned to the biologically important scenario of interferon modulation of NFκB-driven inflammatory responses.

### Type I and II IFNs Enhance NFκB Responsiveness to dsRNA

**Figure 2:**
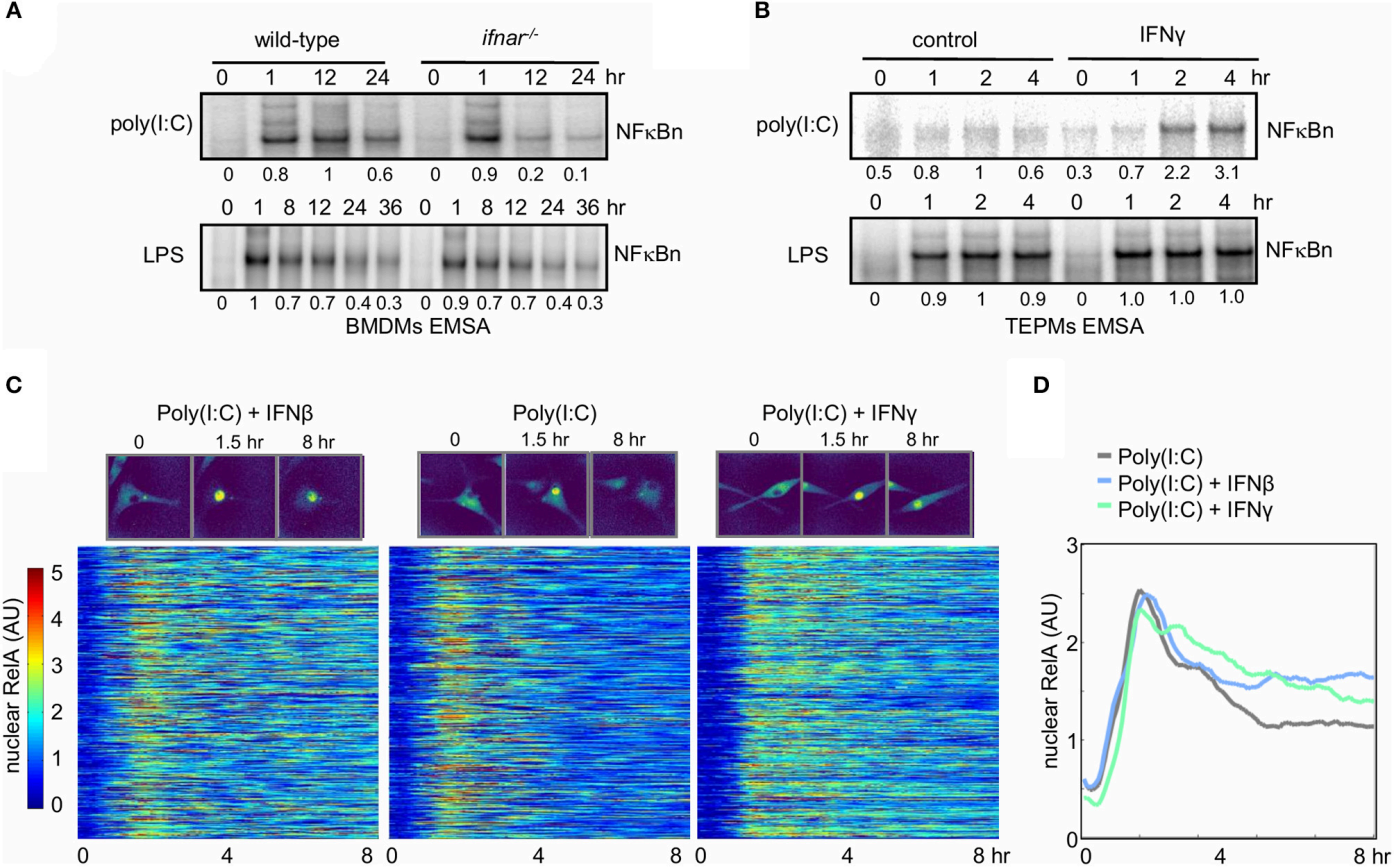
Interferons potentiate NFκB activation in response to the viral PAMP poly(I:C). **(A)** Electrophoretic mobility shift assay (EMSA) of nuclear NFκB activity in wild-type and *ifna*r^−/−^ BMDMs stimulated with LPS and poly(I:C). Quantitated activity is indicated below each band. **(B)** EMSA of nuclear NFκB activity in TEPMs cultured with or without IFNγ for 24 h prior to exposure to poly(I:C) or LPS. **(A,B)** show data representative of three biological replicates. Quantitations of phosphorimager data are relative to peak activity in controls which is set to 1. **(C)** Single-cell tracking of RelA-mVenus localization in 577 Poly(I:C) stimulated BMDMs cultured in the absence or presence (24 h) of IFNβ and IFNγ. Nuclear NFκB activity is indicated as nuclear:cytoplasmic ratio. The time-course response of each tracked cell is displayed as a row in the heatmap with brighter colors corresponding with increasing nuclear localization of NFκB. **(D)** The average nuclear NFκB activity of 577 tracked cells is shown for naïve and IFNβ- and IFNγ-primed conditions. **(C,D)** show data representative of two biological replicates.

Exposure of naïve macrophages to Type I (IFNβ) or Type II (IFNγ) interferons alters their physiological functions and gene expression responses to pathogen-associated molecular patterns (PAMPs) or inflammatory cytokines [reviewed by Glass and Natoli (53); Ivashkiv and Donlin (54); Lawrence and Natoli (55)]. The underlying molecular mechanisms may involve changes to state of the chromatin or epigenome (34), or alterations to the signaling network state. We established two experimental systems to examine whether and how interferon signaling affected the control of NFκB signaling. To determine whether NFκB activity is modulated by Type I Interferon, bone marrow-derived macrophages (BMDMs) from either wild-type or type I interferon receptor-deficient (*ifna*r^−/−^) mice were treated with LPS (sensed by TLR4) or the dsRNA mimetic poly(I:C) (sensed by TLR3, RIG-I, and MDA-5). *Ifna*r^−/−^ macrophages do not sense the tonic or PAMP-responsive production of IFNβ that may be referred to as “IFNβ feedback” (56). Nuclear extracts analyzed by electrophoretic mobility shift assay (EMSA) revealed that in response to LPS NFκB induction was similar between the WT and *ifna*r^−/−^ BMDMs (Figure 2A, lower panel), but in response to poly(I:C) it was similar only at the 1 h timepoint and significantly reduced at later time points in the knockout (0.2 and 0.1 vs. 1.0 and 0.6 relative DNA binding activity, Figure 2A upper panel).

As type II interferon is produced by T-cells and known to polarize naive macrophages to a more activated state, we addressed the role of type II interferon (IFNγ) on NFκB signaling in primary peritoneal macrophages elicited by thioglycollate (TEPMs). Cells were cultured with or without IFNγ for 24 h prior to exposure to poly(I:C) or LPS before we examined the effect of IFNγ priming on NFκB signaling by EMSA. Whereas, IFNγ did not affect LPS-induced NFκB activation, it strongly enhanced the NFκB responsiveness to poly(I:C) at 2 and 4 h (2.2 and 3.1 vs. 1 and 0.6 relative NFκB DNA binding activity, Figure 2B).

Recent single-cell imaging studies have revealed that NFκB nuclear localization dynamics can show diverse single-cell dynamics which can be obscured in bulk assays (49, 57, 58). To quantitatively measure the effects of type I and type II IFN pretreatment on NFκB dynamics BMDMs derived from a RelA-mVenus reporter mouse were stimulated with poly(I:C) and nuclear NFκB translocation was tracked in single cells. Plotting the nuclear NFκB trajectory for 577 cells in each condition, revealed that even in the context of cellular heterogeneity, either interferon (Type I or II) increased nuclear NFκB activation at late timepoints in response to poly(I:C) (Figure 2C). Indeed, the average of these single-cells trajectories confirmed this also (Figure 2D). Total NFκB abundance in response to poly(I:C) did not increase with either IFNβ or IFNγ co-stimulation, indicating increased nuclear NFκB was not due to increase abundance of NFκB protein (Supplementary Figure 3). Given that neither IFNβ nor IFNγ lead to IKK activation (as long as the preparations are endotoxin-free), these results suggest that late NFκB activity in WT macrophages responding to poly(I:C) may be enhanced by conditioning macrophages with type I or II interferon. We hypothesized that IFN-mediated regulation of IκB synthesis and/or free IκB degradation might underlie the observed cross-regulation, and we utilized SiMoN to dissect the mechanism.

### Type I IFN Feedback Amplifies dsRNA-Induced NFκB Activity by Inhibiting IκBα Synthesis

**Figure 3:**
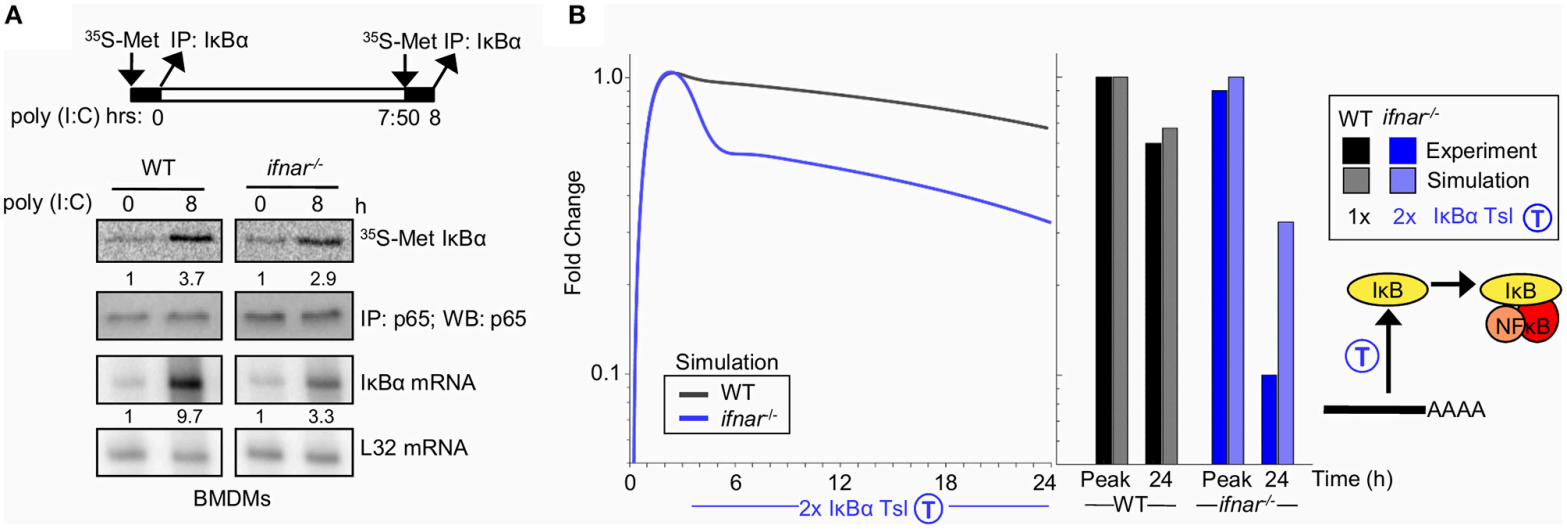
Type I interferon signaling potentiates late NFκB activity by translational inhibition of IκBα **(A)** Experiments to determine IκBα translation rate in BMDMs. Top, schematic of the experimental design: ^35^S-labeled Methionine pulsed at 0 h and following 8 h of Poly(I:C) stimulus. Middle, immunoprecipitates of IκBα following a ^35^S-methionine pulse at either indicated timepoint. NFkB p65 immunoprecipitates are shown as normalization controls. Bottom, IκBα mRNA analysis using RNA protection assay. Ribosomal protein gene L32 is provided as a control. These data are representative of three biological replicates. Quantitations are relative to basal conditions which is set to 1. **(B)** Using SiMoN to determine whether the measured changes in the translation rate are sufficient to account for the NFkB activation defect in *ifna*r^−/−^ BMDMs. Left, timecourse simulation of NFκB activity in response to IKK activation following poly(I:C) stimulation with and without a 2-fold increase in IκBα translation measured in *ifna*r^−/−^ BMDMs **(A)**. Right, bar graph of NFκB activity at the peak and 24 h time point as quantified from the simulation and experiment (Figure 2A). This indicates that the increase in translation rate measured in **(A)** is not sufficient to account for the decrease in NFkB activity observed in Figure 2A.

Type I interferon signaling is known to result in inhibition of the translation of select mRNAs (59). To investigate whether type I interferon feedback alters IκBα translation, we measured IκBα protein synthesis in response to poly(I:C) directly in WT and *ifna*r^−/−^ BMDMs. Following stimulation with poly(I:C) for 8 h, we pulsed with ^35^S-labeled Methionine, and IκBα was immunoprecipitated to examine newly synthesized IκBα levels. Despite significantly lower concentrations of IκBα mRNA template (9.7 vs. 3.3 fold induction, 1.2 ±0.6 log_2_ fold difference based on triplicates), the amounts of ^35^S-Met IκBα levels were similar in WT and *ifna*r^−/−^ BMDMs in response to poly(I:C) (Figure 3A, 3.7 vs. 3.2 fold induction, −0.1 ± 0.4 log_2_ fold change, based on triplicates), indicating that an IFNAR-dependent process inhibits translation during BMDM response to poly(I:C). Indeed, quantitation of the fold induction of synthesis (^35^S-labeled IκBα) over the fold induction of the mRNA level shows that there is a 2-fold higher degree of IκBα translation in the *ifna*r^−/−^ BMDMs than wild-type counterparts (Figure 3A, 1.1 ± 0.72 log_2_ fold change, based on summing the standard deviations in the quadrature). While there is substantial uncertainty in the quantitation of type I IFN-dependent translation inhibition the above-described measurements place the true value between 1 and 4-fold with 2-fold being the geometric mean.

During the early phase of the poly(I:C) timecourse, prior to any potential IFNβ feedback, NFκB activation is equivalent in wild-type and *ifna*r^−/−^ macrophages. However, at later time points that may involve type I IFN feedback signaling, NFκB activation is significantly lower in *ifna*r^−/−^ BMDMs (Figure 2A). To determine whether type I IFN-dependent translation inhibition may account for the defects in NFκB activation in *ifna*r^−/−^ BMDMs, we used SiMoN to quantify the effect of translational inhibition and IKK activity on NFκB activation (Figure 3B). In both WT and *ifna*r^−/−^ BMDMs, TLR3/TRIF signaling triggers IKK and NFκB activity during the early phase. By comparing NFκB activity using SiMoN with and without the addition of a 2-fold increase IκBα translation as identified experimentally in *ifna*r^−/−^ BMDMs we found a qualitative agreement in decreased late-phase NFκB activity (Figure 3B). However, as the simplified model could only explain a 3-fold difference in late-phase NFκB activity, rather than the 6-fold difference observed experimentally, as such our analysis using SiMoN says that for NFκB to remain fully elevated in wild-type cells in response to poly(I:C), translation inhibition alone is not sufficient and an additional mechanism of cross regulation is required. We wondered whether IFNβ may also modulate IKK activity itself in response to poly(I:C)-induced NFκB activity.

### The Type I IFN-Induction of RIG-I Enhances dsRNA-Responsive IKK Activation

**Figure 4:**
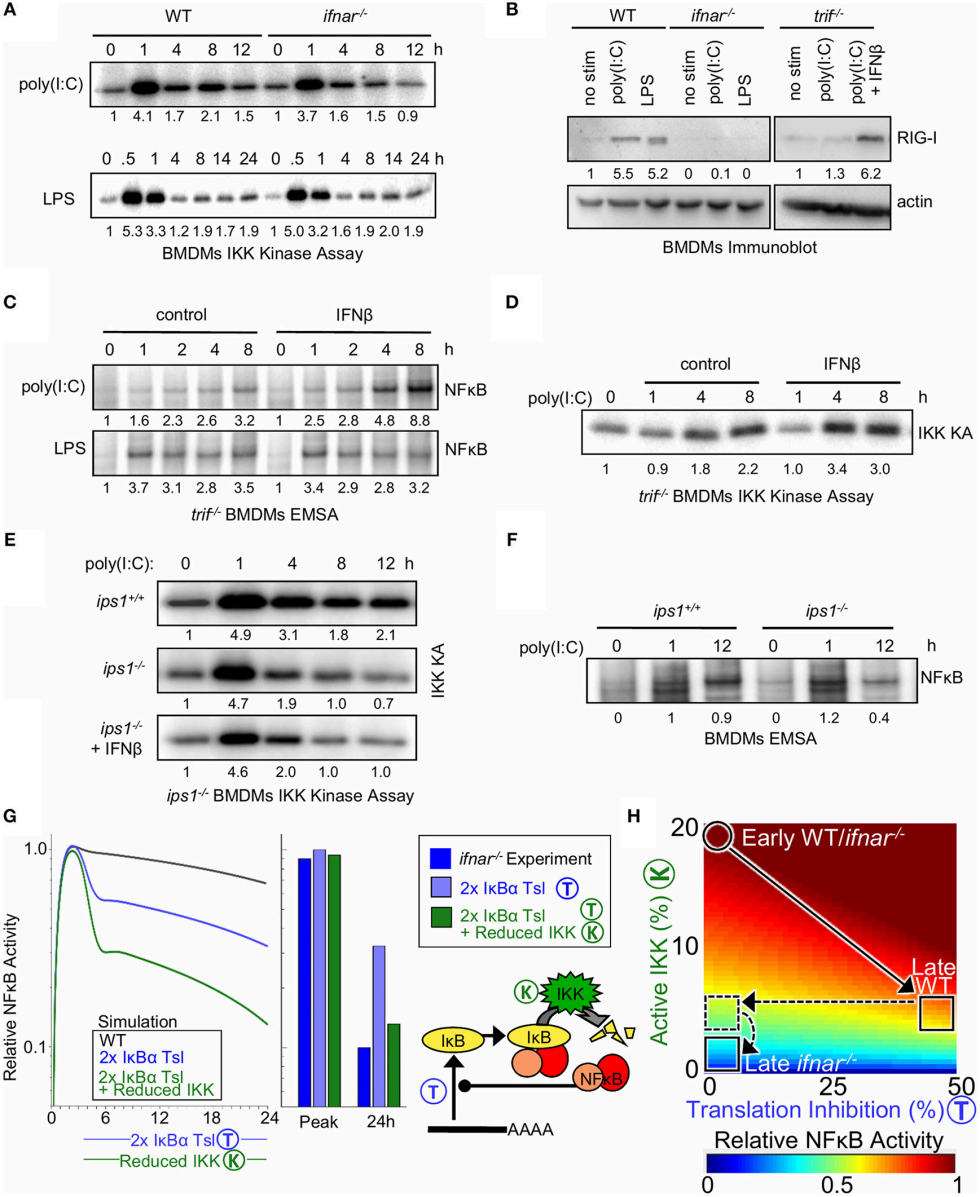
Type I interferon potentiates late NFκB activation by poly(I:C) by decreasing IκB translation and increasing bound IκB degradation via elevated RigI expression. **(A)** Immunoprecipitation kinase assay (kinase A) of IKK activity in WT and *ifna*r^−/−^ BMDMs in response to poly(I:C) and LPS. **(B)** Immunoblot of RIG-I expression after 8 h of poly(I:C) or LPS treatment in WT, *ifna*r^−/−^ and *tri*f^−/−^ BMDMs; and rescue of *tri*f^−/−^ cells with IFNβ. **(C)** EMSAs of NFκB activation by poly(I:C) and LPS in *tri*f^−/−^ BMDMs with and without IFNβ co-treatment. **(D)** IKK activity in WT and *ip*s^−/−^ BMDMs exposed to poly(I:C) and in *ip*s^−/−^ cells with co-treatment with IFNβ. **(E)** IKK activity in *tri*f^−/−^ BMDMs with and without IFNβ co-treatment. **(F)** EMSAs of NFκB activation by poly(I:C) in ips1^+/+^ and *ips1*^−/−^ BMDMs. **(A–D)** show a dataset representative of at least three biological replicates, and **(E,F)** show a representative of two biological replicates (we gratefully acknowledge Zhijian James Chen for *ips1*^−/−^ bone marrow). Quantitations are relative to basal or peak activity, which is set to 1. **(G)** (Left) Simulated NFκB timecourse in response to IKK activation representative of poly(I:C) stimulation, with a 2-fold increase in IκBα translation (blue) or with both IκBα translation inhibition and 50% IKK activity reduction as seen in *ifna*r^−/−^ (green). (Right) Bar graph of NFκB activity at the peak and 24 h time point as quantified from simulations and experiments (Figure 2A). **(H)** Heatmap of NFκB activity calculated using SiMoN for 50 increasing IKK activity values and 50 increasing degrees of translation inhibition (2,500 total points). In both WT and *ifna*r^−/−^ poly(I:C) stimulation results in increased IKK activity during the early phase. Following this WT cells undergo 50% translation inhibition and IKK activity decreases. *ifna*r^−/−^ cells lack translation inhibition (horizontal dashed line, Figure 3), and have decreased late-phase IKK activity [vertical dashed line, this **(A–F)**].

To test the model-generated prediction of an additional molecular mechanism by which type I IFN regulates NFκB activity in response to poly(I:C), IKK activity was examined. In response to poly(I:C), the initial 1 h peak of IKK activity was similar between WT and IFNAR-deficient BMDMs (4.1. vs. 3.7 fold i.e., ≤10% different), yet IKK activity was lower at 8 and 12 h in *ifna*r^−/−^ macrophages (2.1 vs. 1.5 fold at 8 h and 1.5 vs. 0.9 fold at 12 h, i.e., ≥30% different, Figure 4A). In contrast, the IKK activity profiles in response to LPS between WT and *ifna*r^−/−^ BMDMs were similar (1.7 vs. 2.0 fold at 8 h and 1.9 vs. 1.9 fold at 12 h).

Whereas, type I IFN feedback is important for inhibition of IκBα synthesis, the IFN-dependent late-phase IKK activity enhances IκBα degradation in response to poly(I:C). Both LPS and poly(I:C) involve TRIF signaling to IKK and resultant induction of IFNβ; however, the fact that we only observed IFN feedback for potentiated NFκB activation in response to poly(I:C) but not LPS led us to investigate whether a TLR3/TRIF-independent mechanism for IKK activation may be boosted by type I IFN signaling. To determine whether a TLR3/TRIF-independent pathway contributes to late IFN-dependent IKK and NFκB activity, BMDMs from wild-type and *tri*f^−/−^ mice were treated with poly(I:C). As expected, we found that in the absence of TRIF signaling, NFκB and IRF/ISGF3 activation by poly(I:C) is severely diminished (Supplementary Figure 4A). However, while the early NFκB activity at 1 h was completely lost, a small amount of late 8–12 h NFκB activity was still observed in *tri*f^−/−^ BMDMs, pointing to a TRIF-independent mechanism to activate NFκB, one that may be boosted by type I interferon signaling.

We considered that the poly(I:C) added to the extra-cellular medium may be taken up by macrophages to activate intracellular cytoplasmic dsRNA receptors. The cytoplasmic dsRNA receptors MDA5 (melanoma-differentiation-associated gene 5) and RIG-I (retinoic-acid-inducible protein I) are known to activate the IRF3 pathway, as well as the IKK complex (60–62). We observed that RIG-I is inducibly expressed (> 5 fold) after 8 h of poly(I:C) or LPS treatment in an IFNAR-dependent manner (Figure 4B). In *tri*f^−/−^ BMDMs, which are deficient in autocrine IFNβ signaling, co-treatment with IFNβ was required to up-regulate RIG-I expression (6.2 vs. 1.3 fold). In addition, quantifying recent results from Cheng et al. (63) revealed transcriptional upregulation of RIG-I mRNA (Ddx58) in response to IFNβ conditioning (Supplementary Figure 4B). Thus, we hypothesized that complementing *tri*f^−/−^ BMDMs with exogenous IFNβ would enhance NFκB activation by poly(I:C). Indeed, IFNβ co-stimulation of *tri*f^−/−^ BMDMs enhanced induction of NFκB activity in response to poly(I:C) (8.8 vs. 3.2) but not LPS (Figure 4C). Furthermore, poly(I:C)-induced, TRIF-independent IKK activity was enhanced by co-treatment with IFNβ (Figure 4D). Together, these results suggest a model in which type I interferon amplifies poly(I:C)-induced NFκB activation through the expression of the intracellular dsRNA sensor RIG-I or MDA5 (64), which activates the canonical NFκB pathways through IKK.

To test whether poly(I:C) responsive NFκB activation is enhanced by RIG-I in this manner, we examined if IKK and NFκB activation in BMDMs is dependent on the RIG-I/MDA5 signaling adaptor IPS-1 (also known as mitochondrial anti-viral signaling protein, MAVS), which signals to IKK and IRF3 (64). Similar to what we observed in the *ifna*r^−/−^ BMDMs, IKK activation by poly(I:C) in *ips1*^−/−^ BMDMs is dampened at late time points (Figure 4E), suggesting that late poly(I:C) IKK activation is mediated by RIG-I/MDA5. Furthermore, unlike our results from *tri*f^−/−^ BMDMs (Figure 4D), IKK activation cannot be enhanced by co-treatment with IFNβ in the *ips1*^−/−^ macrophages (Figure 4E). Indeed, poly(I:C)-induced NFκB activation in *ips1*^−/−^ BMDMs was lower at 12 h than in wild-type counterparts (Figure 4F) (0.4 vs. 0.9), though not as low as observed in *ifna*r^−/−^ BMDMs (Figure 2A) (0.2 vs. 1).

Our studies revealed two mechanisms by which type I interferon signaling may modulate NFκB activation (Supplementary Figure 4C). We first showed that interferon signaling inhibits translation of IκBα mRNAs (Figure 3A); we then, upon calculating with SiMoN that this alone was not sufficient (Figure 3B), found that type I interferon induces expression of the cytoplasmic receptor RIG-I which signals to canonical IKK (Figure 4B). Inclusion of both translation inhibition (quantified in Figure 3) and interferon-dependent IKK activity (quantified in Figure 4A) into calculations of NFκB activity with SiMoN fully explained the reduced late-phase NFκB activity in *ifna*r^−/−^ cells (Figure 4G), and delineates how these two mechanisms combine to potentiate NFκB activation by poly(I:C) (Figure 4H). Examining the two mechanisms individually, we find that translational inhibition only partially accounts for the increase in NFκB activation and that the experimentally measured reduction in late-phase NFκB activity in *ifna*r^−/−^ can only be explained when the measured translation inhibition is combined with a reduction in IKK activity (Figure 4H).

Interestingly, both mechanisms of crosstalk between type I interferon and NFκB signaling are specific for dsRNA, rather than LPS-triggered NFκB activation, albeit for different reasons (Supplementary Figure 4C). The RIG-I/MDA5-mediated cross-regulation mechanism is specific because these receptors sense dsRNA and not LPS. In contrast, the fact that the translational inhibition mechanism shows specificity for dsRNA-triggered NFκB activation may be explained by a kinetic argument: translational inhibition has a diminished effect on NFκB activation when IKK-mediated IκB degradation is high. Thus, high IKK activity induced by LPS is sufficient to produce substantial NFκB activity and is only marginally enhanced by interferon-mediated IκBα translational inhibition.

### IFNγ Potentiates NFκB Activation by Enhancing Free IκBα Degradation

**Figure 5:**
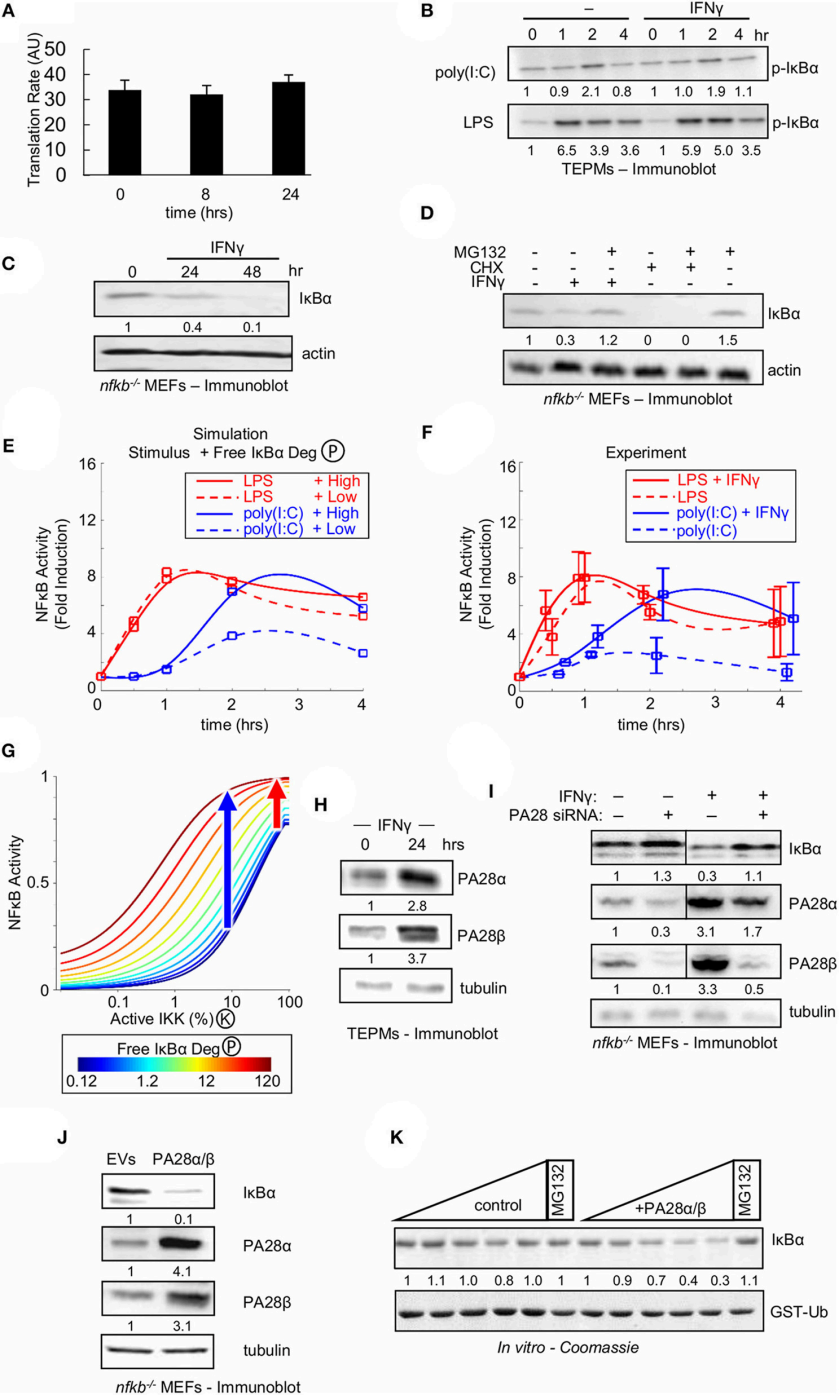
Type II interferon amplifies weak NFκB activating stimuli by enhancing free IκBα degradation. **(A)** IκBα translational synthesis rates in naïve and IFNγ-conditioned TEPMs as revealed by ^35^S-Met pulse assay. Average and standard deviation of three biological replicates are shown. **(B)** Immunoblot for p-IκBα in TEPMs exposed to either LPS or poly(I:C) with or without IFNγ priming. **(C)** Immunoblot of “free” IκBα compared to an actin control in MEFs deficient in canonical NFκB proteins RelA, cRel and p50 (termed “*nfk*b^−/−^”) exposed to IFNγ. **(D)** Free IκBα levels in *nfk*b^−/−^ MEFs compared to an actin control. Immunoblot of lysates produced from MEFs exposed to 24 h priming with IFNγ or 4 h treatment with ribosomal inhibitor CHX, and followed by addition of proteasome inhibitor MG132. **(E)** Predictions from the Simplified Model of NFκB (SiMoN) with low (10% at peak) IKK activity, representative of poly(I:C) (blue), and high (40% at peak) IKK activity, representative of LPS. Values were calculated at 0, 0.5, 1, 2, and 4 h and fit with a smoothing spline for consistency with experimental time points. Free IκBα degradation was modulated from the default value (dashed lines) to 10-fold higher (solid lines) based on quantification of immunoblot in 5B. **(F)** Time course of NFκB induction (quantitated from EMSAs) in naïve or IFNγ-conditioned TEPMs stimulated with poly(I:C) and LPS. **(G)** Nuclear NFκB activity calculated using SiMoN as a function of bound IκBα degradation (IKK-activity, x-axis) and free IκBα degradation (colored lines). The blue and red arrows indicates the free IκBα degradation-dependent increase in NFκB activity for low and high IKK activities indicative of poly(I:C) and LPS, respectively. **(H)** Immunoblots of proteasome activator 28 (PA28) levels in TEPMs following exposure to IFNγ. **(I)** Immunoblots for IκBα and proteasome activator 28 (PA28) in *nfk*b^−/−^ MEFs. Both conditions were repeated following PA28 siRNA-mediated knockdown. **(J)** Immunoblot of IκBα and PA28 levels in *nfk*b^−/−^ MEFs transduced with retroviral transgenes. **(K)** Coomassie-stained SDS-PAGE showing free IκBα and PA28α/β levels following incubation with increasing amounts of purified 20S proteasome (upper panel) contrasted with GST-ubiquitin levels (lower panel), which serves as a negative control. **(B–D)** show a dataset representative of at least three biological replicates **(H–K)** show a dataset representative of two biological replicates. Quantitations are relative to basal or *t* = 0 activity, which is set to 1.

Akin to type I interferon signaling in BMDMs, paracrine type II interferon used for priming TEPMs enhances nuclear NFκB DNA binding activity in response to poly(I:C) stimulation more than 2-fold, whereas it had little effect on LPS-induced NFκB activation (Figure 2B). To investigate the mechanism by which IFNγ potentiates NFκB responsiveness to poly(I:C) we again quantitatively examined the three tunable reactions controlling IκB metabolism using SiMoN (Figure 1). Specifically, we wondered whether IκB translation is inhibited in a Type II IFN-dependent manner in addition to the Type I-dependent inhibition we identified. However, we found no evidence that IFNγ treatment affects mRNA translation rates when translation rates were measured using the ^35^S-Met pulse experiment (Figure 5A). Next, we tested whether IFNγ alters the IKK activity profile induced by poly(I:C) or LPS. To our surprise, IFNγ pre-treatment did not alter LPS- or poly(I:C)-induced IKK activity (Figure 5B).

As two out of the three reactions represented in SiMoN were found unaffected by IFNγ we tested the third, the degradation rate of unbound IκBα. Whereas, NFκB-bound IκBα is degraded through IKK-mediated phosphorylation and the ubiquitin-proteasome system, free IκBα is degraded independently of IKK activity through a ubiquitin-independent, but 20S proteasome-dependent mechanism (65, 66). To determine whether IFNγ affects the stability of free IκBα, we employed MEFs deficient in the NFκB proteins RelA, cRel, and p50 (termed “*nfk*b^−/−^”) in which all IκBα is in fact free, a previously established assay system for free IκBα turnover (51): *nfk*b^−/−^ cells were treated with IFNγ, and IκBα levels were measured by Western blotting. IFNγ treatment of *nfk*b^−/−^ cells resulted in a reduction of cellular IκBα (Figure 5C). We next sought to confirm that IFNγ-mediated reduction of free IκBα was due to enhanced degradation rather than reduced synthesis. We found that addition of the proteasome inhibitor MG132 after 24 h of IFNγ rescued the IκBα level, whereas addition of MG132 to cells treated for 4 h with the ribosomal inhibitor CHX did not (Figure 5D). Together, these data suggest that IFNγ enhances the proteasomal degradation of free IκBα.

We employed SiMoN to determine whether enhanced degradation of free IκB protein may account for the experimentally observed IFNγ-potentiated NFκB activity in response to poly(I:C). Our Western blot analysis is consistent with 10-fold higher degradation in IFNγ-primed cells; using this number in simulations along with low and high IKK activity curves representative of poly(I:C) and LPS, respectively, resulted in more than 2-fold amplification of NFκB activation in response to weak IKK activator poly(I:C) (Figure 5E). SiMoN predicted that increased free IκBα degradation affected the NFκB response speed, but did not substantially change late (>1 h) NFκB activity to strong IKK activating signals such as LPS but greatly increased the NFκB activity to weak activating signals such as poly(I:C) (Figure 5E). Strikingly, these predictions were validated by experimental quantitation of NFκB fold induction, which demonstrated similarly selective amplification of poly(I:C) but not LPS (Figure 5F). To understand this selective amplification we used SiMoN to quantify the relationship between IKK activity and NFκB and how this dose-response relationship is altered by free IκBα degradation. We observed a shift in the dose-response relationship between NFκB and IKK activities with increasing free IκBα degradation (Figure 5G). This shift selectively amplifies the NFκB response to weaker IKK-activating stimuli without substantially affecting strong IKK activators. Thus, the specificity of IFNγ-mediated potentiation of NFκB activation for poly(I:C), but not LPS, may be sufficiently explained by a kinetic argument: namely, weak signals are subject to modulation by crosstalk mechanisms, whereas strong signals are less sensitive to such modulation.

### The IFNγ-Induced PA28 Proteasome Activators Accelerate Free IκBα Degradation

As IFNγ-stimulated degradation of free IκBα may tune NFκB responsiveness to poly(I:C) in tissue resident macrophages, we considered the potential molecular mechanisms. SiMoN predicts the molecular mechanism need not be poly(I:C) specific as selective amplification of weak NFκB activators can emerge through the kinetics of non-specific increased degradation of free IκBα. Whereas, ubiquitinated proteins are recognized and degraded by the 26S proteasome, which consists of the 20S barrel-shaped core and a 19S regulatory cap, free IκBα was shown to be degraded in a ubiquitin-independent manner (65). An alternative 11S regulatory cap, consisting of oligomers of the PA28α and PA28β proteins allows for ubiquitin-independent entry into the proteasome and has been implicated in antigen processing in antigen-presenting cells (66, 67).

Western-blotting revealed that IFNγ treatment increased PA28α and PA28β expression in both TEPMs (Figure 5H) and MEFs (Figure 5I). Using *nfk*b^−/−^ MEFs allowed us to assay expression of free IκB protein, and examine whether PA28-mediated proteasomal degradation controls free IκB abundance. Knockdown of PA28α and PA28β by siRNA in *nfk*b^−/−^ MEFs resulted in increased IκBα levels in cells, particularly in cells exposed to IFNγ (Figure 5I). Conversely, stable retroviral overexpression of PA28α and PA28β in *nfk*b^−/−^ MEFs led to decreased levels of free IκBα (Figure 5J), demonstrating that increased expression of PA28α and PA28β are sufficient to increase degradation of free IκBα. Taken together, these data suggest that the 11S proteasomal cap components PA28α and PA28β are necessary and sufficient to increase free IκBα degradation in IFNγ-primed cells.

To further demonstrate a direct role for the IFNγ-inducible PA28 proteins in free IκBα degradation, purified IκBα was subjected to an *in vitro* degradation assay with purified 20S proteasome. The presence of PA28 proteins accelerated the degradation of IκBα in this cell-free system (Figure 5K, upper), and this finding was specific to IκBα as the use of ubiquitin as the substrate in the same assay showed no change upon addition of PA28 proteins (Figure 5K, lower).

### IFNγ-Mediated Degradation of Free IκBα Sensitizes NFκB to Weak Activating Signals

Our studies revealed that type II interferon signaling amplifies NFκB activation through increasing free IκBα degradation (Supplementary Figure 5A). SiMoN predicts that the amplifying effect of increasing free IκBα degradation is not specific to poly(I:C), but general to other weak NFκB inducing signals (Figure 5G). To further validate this prediction we utilized UV radiation, a known weak activator of NFκB, causing translation inhibition that allows for depletion of IκBα through its constitutive turnover (68). Consistent with the model predictions, pretreatment with IFNγ increased the NFκB response to UV in wild-type immortalized MEFs (Supplementary Figure 5B).

SiMoN was used to simulate the unfolded protein response (UPR) (69) which increases free IκBα degradation rates (simulating the presence of IFNγ). Whereas, increasing the free IκBα degradation rate had little effect on the response to large IKK activity changes such as for LPS (Figure 5G), it is predicted to result in a significant increase in the peak of NFκB activity in response to UPR (Supplementary Figure 5C).

To test this prediction and establish whether increased expression of PA28α and PA28β is sufficient to alter NFκB responsiveness to UPR, wild-type MEFs were retrovirally transduced with PA28α and PA28β. Overexpression of PA28α and PA28β increased the NFκB response to UPR induced by thapsigargin (Supplementary Figure 5D). The NFκB response to the strong IKK activator, TNF, however, was unaffected by the overexpression of PA28α and PA28β, consistent with the computational prediction that stronger inducers of IKK activity are not sensitive to increased free IκBα degradation (Figure 5G and Supplementary Figure 5D). In addition, pa28-deficient MEFs showed reduced response to thapsigargin (Supplementary Figure 5E). In addition, quantifying recent results from Cheng et al. (63) revealed transcriptional upregulation of PA28α/β (Psme1/2) in response to IFNγ conditioning (Supplementary Figure 5F). Together, these data support a model in which IFNγ enhances NFκB responses to weak stimuli by increasing the IKK-independent degradation of free IκBα via enhancement of the 11S proteasomal degradation pathway.

## Discussion

Here we presented a new simplified mathematical model of NFκB activity (SiMoN) and applied it to studying how interferons modulate NFκB activity. Although this model lacks the some of the molecular network detail of other NFκB signaling models that describe the highly dynamic and variable NFκB responses at single cell resolution (10), it provides for an intuitive understanding of how NFκB is controlled at the tissue scale. Specifically, the abstraction revealed that NFκB activity is governed fundamentally by three reactions that may be modulated by signaling crosstalk. This is an important modification of the prevailing research focus on just one of these: the IKK-controlled degradation of NFκB-bound IκB. Our work demonstrates that a focus on IKK alone has substantially limited previous studies into mechanisms of signaling crosstalk by cytokines that themselves do not activate NFκB. in this manner it is important to point out that other mechanisms that do not affect IκB metabolism may also control NFκB activity (e.g., the nuclear import/export machinery, post-translational modifications of NFκB, and expression of NFκB protein family members) and could be included in further studies.

In response to infection, innate immune responses must be delicately coordinated to ensure that it is sufficient to mount an effective defense, but not excessive so as to avoid the potentially harmful effects of inflammation. A central regulator of this response is NFκB, which can be activated by a variety of pathogen sensors, such as RIG-I/TLR3 and TLR4 in response to viral RNA and bacterial LPS, respectively. Infections also trigger an upregulation of type I interferon expression and expression of type II IFNγ by T and NK cells, thus providing a variety of cytokine milieus that potentially affect the NFκB-driven immune response. We have shown here how both type I and type II interferons engage in signaling crosstalk with the core of IκB metabolism, effecting a stimulus-specific potentiation of NFκB activation, yet do so via different molecular mechanisms.

**Figure 6:**
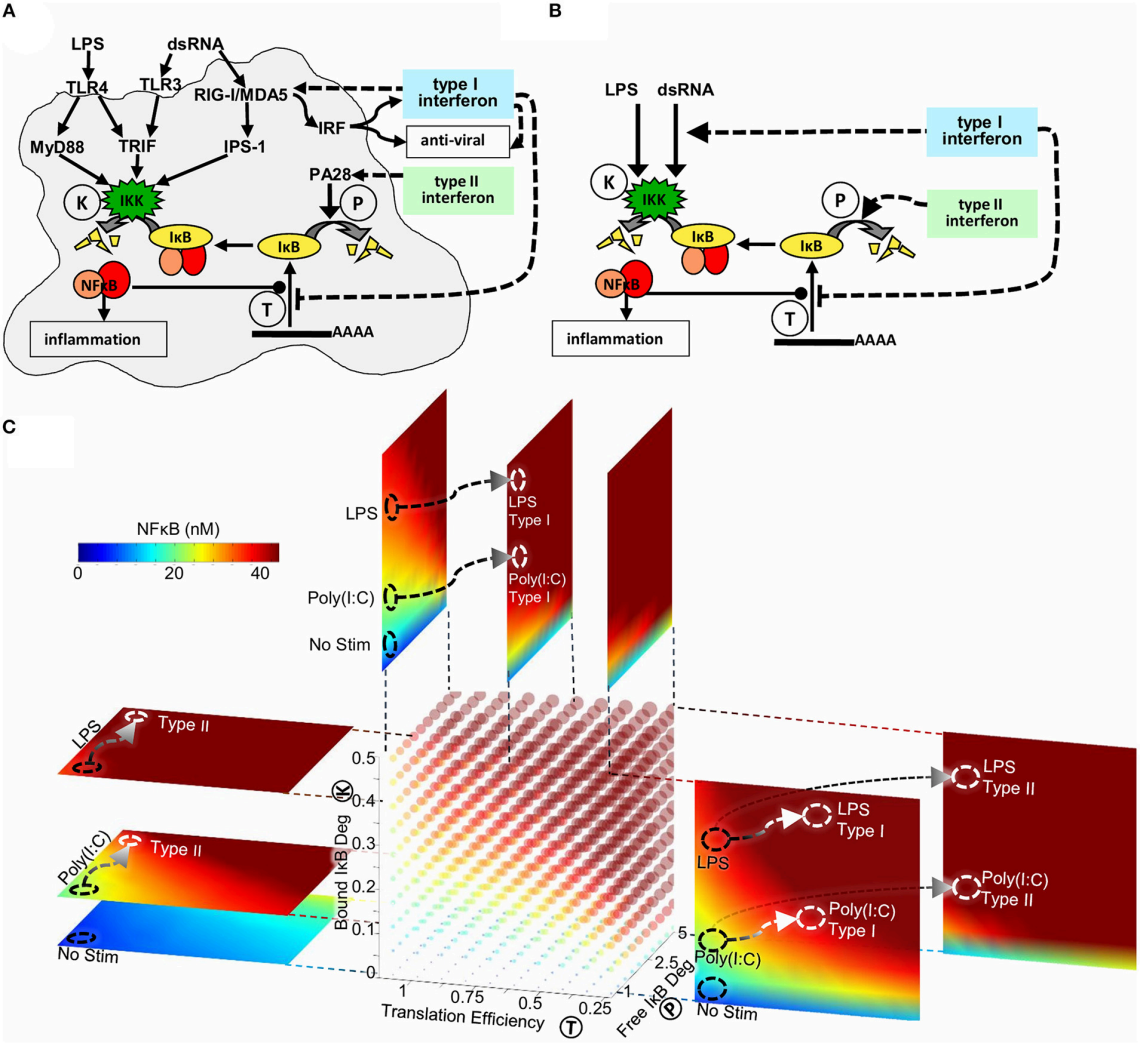
The mechanisms underlying interferon signaling crosstalk on NFκB. **(A)** Type I interferons reduce IκB expression and increase IKK activity through RIG-I and IPS-1. Type II interferons increase free IκB degradation through a PA28-dependent process. **(B)** Type I interferons reduce translation of IκBα and increase the expression of the cytosolic viral sensor to allow for enhanced IKK mediated degradation of NFκB-bound IκBα. Type II interferon increases the degradation rate of free IκBα. All mechanisms potentiate the NFκB response to weak signals emanating from viral PAMP sensors, but have little effect on bacterial-MyD88-mediated responses. **(C)** Three-dimensional heatmap of nuclear NFκB concentrations as a function of three biochemical reactions: IKK activity (reaction K), IκB translation efficiency (reaction T) and free IκB degradation (reaction P). The point in this parameter space reached following Poly(I:C) and LPS stimulus is marked with black circles. Signaling crosstalk by Type I and Type II interferons produce distinct trajectories through this three-dimensional parameter space (marked with white arrows to white circles).

Through quantitative analysis of experimental data using SiMoN we identified two reactions in the core NFκB signaling module that are modulated by type I interferon feedback. Reduction in IκBα translation inhibition combined with modulation of IKK activity through RIG-I/MDA5 and IPS-1 results in increased late stage NFκB activation in response to poly(I:C) (Figure 6A). Type II interferon priming was found to modulate a third reaction, that we had not tested in response to type I interferon. Namely, type II interferon increased free IκBα degradation via the induction of immunoproteasomal cap proteins, thereby amplifying NFκB activation in response to weakly activating stimuli such as poly(I:C) (Figure 6A). IFNγ exposure also amplified the NFκB in response to ribotoxic stimuli, such as UPR, which induces NFκB signaling without inducing IKK (Supplementary Figure 5C), but showed less effect on LPS which activates NFκB by strongly inducing IKK.

Interestingly, the selective amplification of low IKK activating signals by IFNγ can be intuitively seen by studying the analytical solution to SiMoN. By first investigating a scenario without free IκB degradation such that the term P tends toward 0 we obtain:

$$\begin{array}{l} \underset{p\to 0}{\lim}\;\frac{0.1{\text{k}}_{\text{f}}\text{K }+\;\sqrt{{\left(0.1{\text{k}}_{\text{f}}\text{K }-\text{ PK}\right)}^{2}\;+\;0.4\text{PK}({\text{Tk}}_{\text{f}}\;+\;{\text{Kk}}_{\text{f}})}\;-\text{PK}}{2{\text{k}}_{\text{f}}(\text{T }+\text{ K})}\;=\\ \;\frac{0.1{\text{k}}_{\text{f}}\text{K }+\;0.1{\text{k}}_{\text{f}}\text{K}}{2{\text{k}}_{\text{f}}(\text{T }+\text{ K})}\;=\;\frac{0.1\text{K}}{\left(\text{T }+\text{ K}\right)} \end{array}$$

For a weak IKK activating stimulus (*K* = 6% · *k*_*ikk*_) SiMoN gives ~0.028μM of NFκB activity and for strong IKK activators (*K* = 60% · *k*_*ikk*_) SiMoN gives ~0.078μM of NFκB activity (*T* = 0.055 min^- 1^ throughout). In contrast if we investigate the effect of enhancing free IκB degradation such that P is high we see that the limit does not depend on IKK activity:

$$\begin{array}{l} \underset{p\to 0}{\lim}\;\frac{0.1{\text{k}}_{\text{f}}\text{K }+\;\sqrt{{\left(0.1{\text{k}}_{\text{f}}\text{K }-\text{ PK}\right)}^{2}\;+\;0.4\text{PK}({\text{Tk}}_{\text{f}}\;+\;{\text{Kk}}_{\text{f}})}\;-\text{PK}}{2{\text{k}}_{\text{f}}(\text{T }+\text{ K})}\;=0.1\mu \text{M} \end{array}$$

Therefore, the analytical solution reveals that free IκBα degradation can amplify NFκB activity in response to weak IKK activating over 3.5-fold (0.028 to 0.1 μM), but for strong IKK activating stimuli the amplification is far less substantial, with only around a 28% increase (from 0.078 to 0.1 μM).

Whether a stimulus is weak or strong depends on both dose and the pathways dose response. As LPS activation of NFκB is largely governed by the ultra-sensitive MyD88 pathway (49), LPS typically activates IKK strongly (or not at all). PolyIC on the other hand relies on the TRIF pathway, which, in macrophages, activates IKK more weakly. Thus, the crosstalk mechanisms identified here allow type I and type II interferons to potentiate NFκB activity in cells exposed to viral RNA, and less so when exposed bacterial LPS. Given the importance of coordinating innate immune defenses of localized macrophages, and system-wide adaptive immune responses during to viral infection, we suggest that the molecular mechanisms of interferon-NFκB crosstalk described here have pathophysiological relevance particularly where interferon signaling and inflammation are linked such as chronic inflammatory diseases and cancer (70, 71). By rigorously quantifying NFκB activation and IFN in physiological conditions, SiMoN may be used to explain seemingly conflicting physiological observations. For example, while greater inflammation is seen in leishmaniasis when the host IFN response is induced by parasites harboring Leishmania RNA virus (LRV) (28, 29) others have found TLR4 mediated NFκB activation to be unaffected by IFNγ (34). The selective IFN-dependent amplification of NFκB activity discovered here may reveal why some inflammatory conditions are susceptible to IFN-mediated crosstalk while others are not. Further work is required to quantify the degree of NFκB activation in diverse physiological conditions.

Further work is required to quantify the impact of selective amplification of NFκB activity on NFκB-target gene expression. A number of factors make such a task difficult, including gene-specific combinatorial control of NFκB-target genes in combination with other transcription factors interferon-regulatory factors [IRFs and STATs, Cheng et al. (72)]. Recent work has also identified highly gene-specific effects of interferons on chromatin accessibility and as such even genes lacking interferon responsive elements (IREs) may be subject to complex crosstalk (63). Similar signaling crosstalk may affect transcriptional elongation, mRNA processing and turnover. Disentangling these effects will require careful quantitative consideration, perhaps with the aid of a quantitative model of the mechanism controlling mature mRNA abundance.

The simplified model presented here enabled an analytical solution for the quasi-steady-state concentration of NFκB as a function of bound IKK activity, free IκB degradation, and IκB translation affinity (Figures 1B, 6B). NFκB activity can thus be calculated when the values of these parameters are known, without the need for timecourse simulations. This has enabled us to make SiMoN available through a web interface (signalingsystems.ucla.edu/tools/SiMoN.html) to allow others to interpret the impact of perturbations in these core processes on NFκB activity. Indeed, NFκB activity may be visualized in a four-dimensional plot (color cube) as a function of the three reactions (Figure 6C). Slices of the color cube in any of the three dimensions reveal NFκB activity as a function of two of the reactions at specific values of the third reaction. Thus, within a single image NFκB activity can be related to the activity of three interferon-tunable reactions that control IκB synthesis and degradation.

## Materials and Methods

### Mathematical Modeling

A new mathematical model was constructed that consists of three ordinary differential equations (ODEs) to describe NFκB activation in response to TLRs and enable studies of signaling crosstalk in cell populations. NFκB activity is a function of its interaction with IκB, whose abundance is controlled via NFκB-dependent synthesis and two degradation reactions (51).

(1) $$\begin{array}{l} \frac{dNF\text{κ}B}{dt}=-k_{f}\;\cdot \;\left[NF\text{κ}B\right]\;\cdot \;\left[I\text{κ}B\right]\\ \;+k_{ikk}\;\cdot \;ikkActivity\;\cdot \;[NF\text{κ}B-IB] \end{array}$$

(2) $$\begin{array}{l} \frac{dI\text{κ}B}{dt}=-k_{f}\cdot \;[NF\text{κ}B]\;\cdot \;[I\text{κ}B]+\;k_{I\text{κ}BExp}\;\cdot \;[NF\text{κ}B]\\ \;-k_{I\text{κ}BDeg}\;\cdot \;[I\text{κ}B] \end{array}$$

(3) $$\begin{array}{l} \frac{dNF\text{κ}B-I\text{κ}B}{dt}\;=\;+k_{f}\cdot \;[NF\text{κ}B]\;\cdot \;[I\text{κ}B]\\ \;-k_{ikk}\cdot [NF\text{κ}B-I\text{κ}B] \end{array}$$

All parameters were derived from the existing cellular model of NFκB regulation (12) as follows: 
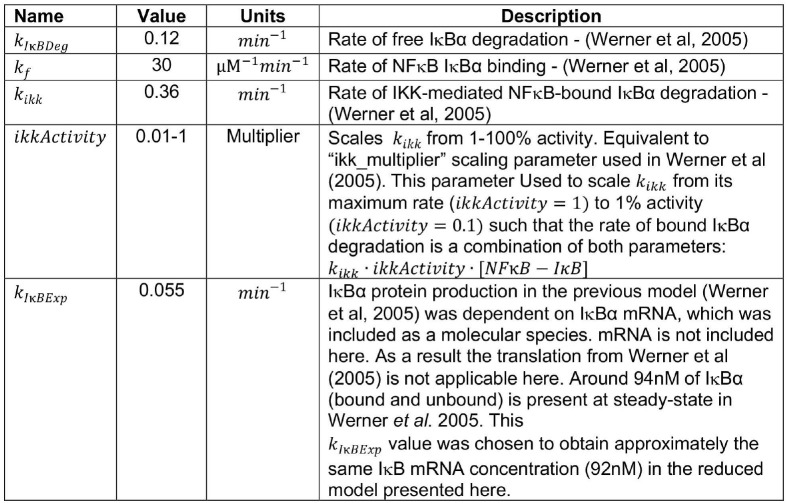


Model construction and analysis was performed in COPASI:Biochemical System Simulator (73). When compared to the model of Werner et al. (12) from which it was derived, SiMoN reduces complexity by assuming all reactions are in a single cellular compartment with all unbound (12) NFκB assumed to be transcriptionally active. Indeed, the majority of inhibited NFκB is found in the cytoplasm with free NFκB quickly translocating to the nucleus in both experimental and model systems (10). In addition, only the predominant NFκB-inhibitor (IκBα) is considered, and IκBε and IκBβ, which are bind a relatively minor portion of NFκB are ignored (74). To further simplify the model, the two reactions of NFκB-dependent IκBα mRNA expression and subsequent protein synthesis are reduced to a single NFκB-dependent IκBα protein production reaction in SiMoN, similar to other reduced models (15–18).

For the exploratory analysis in Figure 1, a steady-state phase was run with default parameters and then initial conditions were updated to the final concentrations from the steady-state phase. The indicated parameters were then scanned using the “Parameter Scan” task in COPASI with a 3-h time course. *k*_*IκBExp*_ was scanned from 0.5 to 1x the default parameter value with samples every 0.1 (Figure 1A), *k*_*IκBDeg*_ was scanned from 1 to 5x the default parameter value with samples every 1 (Figure 1B) and the *ikkActivity* multiplier was scanned from 1 to 2x the default parameters with samples every 0.25 (Figure 1C). Two dimensional parameter scans were performed using nested parameter scan tasks in COPASI to repeatedly perform a steady-state analysis at each parameter value as indicated (Figure 1D). In order to quantify the effect of IκBα translation on Poly(I:C) responses (Figure 3B) the model was modified to add an additional modifier to the rate of IκBα expression (IκBα expression = *k*_*Iκ*_*BExp*·tslModifier, *tslModifier* = 1). A Copasi event was added to trigger at 200 min updating the translation rate modifier parameter, and a parameter scan task in Copasi was used to scan this modifier at 1 (no change) and 2 (double IκBα expression). IKK activity dynamics were simulated by modulating the multiplier of NFκB-bound IκB degradation reaction (parameter *ikkActivity*). Input curves for and poly(I:C)-induced IKK activity (Figure 3A) were quantified using ImageJ software (75). A piecewise function, which interpolated between the time points in the figure, was created to represent IKK activity through modulating the multiplier of NFκB-bound IκB degradation reaction (parameter *ikkActivity*).

In order to simulate the modulation of IKK activity *ifna*r^−/−^ (Figure 4G) two additional multipliers were added scaling early IKK activity (0–200 min) and late IKK activity (>200 min) and these were set to 0.9 and 0.6, respectively to represent the fold change in IKK activity measured in *ifna*r^−/−^ BMDMs by IKK kinase assay (Figure 4A). Simulations of the effect of free IκBα degradation on Poly(I:C) and LPS responses (Figures 5E,F) were obtained by multiplying the *ikkActivity* by 0.5 for Poly(I:C) and 2 for LPS to give peak IKK activity at ~7% for Poly(I:C) and ~30% for LPS, and a parameters scan task was use to adjust *k*_*IκBDeg*_ to 12 $\overset{-1}{\min}\;$for both input curves. Simulations of the unfolded protein response (Supplementary Figure 5C) involved applying, at time *t* = 0, a 50% reduction on the IκB translation rate, while keeping the NFκB-bound IκB degradation reaction rate (dependent on IKK activity) constant at its basal level. In the analytical analysis and figures parameters are abbreviated: *ikkActivity* ·*k*_*ikk*_ = K, *k*_*IκBDeg*_ = *P, k*_*IκBExp*_ = *T*.

### Mouse Strains and Cell Culture

Bone Marrow-Derived Macrophages (BMDMs) were generated from C57BL/6, *tri*f^−/−^, *ips1*^−/−^, and *ifnar*^−/−^ mice with L929 cell–conditioned medium for 8 days. Thioglycollate Elicited Peritoneal Macrophages (TEPMs) were isolated from the peritoneal cavity 4 days after injection of thioglycollate. Mouse Embryonic Fibroblasts (MEFs) of indicated genotype (wild-type or *nfkb1*^−/−^*cre*l^−/−^*rel*a^−/−^) were prepared from embryonic day 12 to 14 embryos and were cultured in Dulbecco's Modified Eagle's Medium (DMEM) containing 10% bovine calf serum for up to six passages. Cells were stimulated with LPS (0.1 μg/ml; Sigma, B5:055), poly(I:C) (50 μg/ml: Amersham Biosciences), IFNβ (250 U/ml: PBL Biomedical Laboratories), IFNγ (eBioscience: 10 U/ml), or thapsigargin (Sigma-Aldrich). For siRNA, the target sequences for PA28α and PA28β were AAGCCAAGGTGGATGTGTT and AGCGAGCAAGGCCAGAAGC, respectively. Oligonucleotides were transfected into *nfkb1*^−/−^*cre*l^−/−^*rel*a^−/−^ MEFs with lipofectamine. This study was carried out in accordance with the principles of the Basel Declaration and recommendations of Association for Assessment and Accreditation of Laboratory Animal Care International (AAALAC) which accredits UCLA's animal care program. UCLA's Animal Welfare Assurance number with the Department of Health and Human Services Office of Laboratory Animal Welfare is A3196-01. The protocol was approved by the UCLA Institutional Animal Care and Use Committee, known as the Chancellor's Animal Research Committee (ARC).

### Live Cell Imaging of NFκB Localization

BMDMs derived from a RelA-mVenus reporter mouse (to be described) were plated on eight-well μ-slides (ibidi) and stimulated with poly(I:C) without or with IFNβ or IFNγ. Conditions were maintained at 5% CO_2_ and 37°c throughout imaging with a Zeiss AxioObserver using a 40x oil immersion objective, LED (light-emitting diode) fluorescence excitation, and CoolSnap HQ2 camera. RelA-YFP and H2B-mCherry images were collected every 5 min over 12 h and exported into MATLAB where analysis was performed as previously described (76).

### Biochemical Assays With Cell Extracts

Nuclear extracts from BMDMs were prepared by high salt extraction. Western blotting analysis and Electrophoretic Mobility Shift Assays (EMSAs) were conducted with standard methods as described previously (12, 46, 68). The κB EMSA probe was: GCTACAAGGGACTTTCCGCTGGGGACTTTCCAGGGAGG. For Western blotting analysis and supershift assays we used antibodies against p65 (Santa Cruz Biotechnology, sc-372), p50 (sc-114), α-tubulin (sc-5286), p50 (N. Rice, NC-1263), lamin A/C (Cell Signaling, #2032), PA28α/β (Cell Signaling, #2408/2409), and IRF3 (Cell Signaling, #4962); Guinea pig anti-RIG-I was used as described previously (77). IKK activity assays were previously described (68). *In vivo* pulse labeling of BMDMs was done with 100 μCi/ml trans ^35^S-Met label (MP Biomedicals, Inc.) using the indicated time courses. IκBα was immunoprecipitated (sc-371) and proteins were resolved on 8% SDS-PAGE, visualized by autoradiography, and quantified with Imagequant software. Ribosomal inhibitor cyclohexamide (CHX) and proteasomal inhibitor MG132 were used to block protein synthesis and degradation, respectively, and as described previously (69). Gene expression studies employed quantitative RNAse protection or qPCR assays, as described (78, 79). Quantitative data of biological replicates was analyzed with indicated statistical tests and visualized in R, Prism, or Excel software.

### Proteasomal *in vitro* Degradation Assay

As previously described (80), 20S proteasome particles were purified from bovine blood using four chromatographic steps (Q-sepharose, Sephacryl S-300, Phenyl sepharose and Mono-Q). PA28α and β subunits were expressed *in E. coli* and purified separately followed by hetero complex formation by refolding following the method described by Song et al. (81). PA28αβ was mixed in 4-fold molar excess with 20S at 25°C, and the resulting proteasome complex was incubated with recombinant IκBα immediately following its elution from a Superdex 200 column. Recombinant IκBα was mixed in varying molar ratios with purified proteasome in a reaction buffer containing 200 mM NaCl, 20 mM Tris HCl, pH 7.1, 10 mM MgCl2, and 1 mM DTT and incubated at 25°C. The reaction was quenched by the addition of 4X SDS dye and boiling for 1 min at 95°C. The products were then separated by SDS-PAGE and visualized by Coomassie staining. To ensure the specificity of the degradative activity of the proteasome the degradation assay was also performed using stably folded GST tagged di-ubiquitin (GST-diUb).

## Author Contributions

Computational modeling was performed by SM. Experiments were performed by EM, JH, and AA. AH conceived the project and AH, SM, and GG designed and coordinated the study. The manuscript was prepared by SM, EM, and AH with contributions from AA, QC, and GG. All authors interpreted the results.

### Conflict of Interest Statement

The authors declare that the research was conducted in the absence of any commercial or financial relationships that could be construed as a potential conflict of interest.
