# Supplementary material for: An NFκB Activity Calculator to Delineate Signaling Crosstalk: Type I and II Interferons Enhance NFκB via Distinct Mechanisms
Source: Front Immunol. 2019 Jun 25;10:1425. doi: 10.3389/fimmu.2019.01425 (PMC6604663; doi:10.3389/fimmu.2019.01425)
Supplement: Supplementary file 1 [file Data_Sheet_1.PDF]

Supplementary Figures

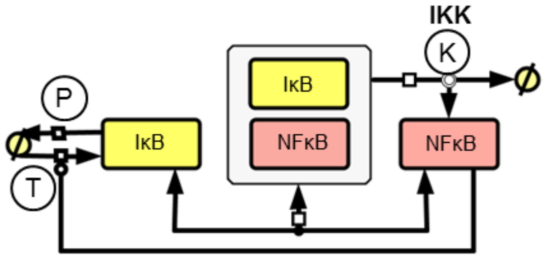

Supplementary Figure 1. The Simplified Model of NFκB (SiMoN) represented in Systems Biology Graphical Notation (Le Novere et al, 2009). The model consists of 3 ordinary differential equations (ODEs) representing mass action kinetics. ODEs are included for IκB, NFκB-IκB complex and NFκB. IKK is not explicitly included as a metabolite but its activity is reflected in the rate of NFκB-bound IκB degradation. Arrows represent reactions which modify the rates of change of each ODE. IκB expression (T) and proteasomal degradation (P) are simulated along with IκB-NFκB binding and IKK-dependent NFκB-bound IκB degradation (K).

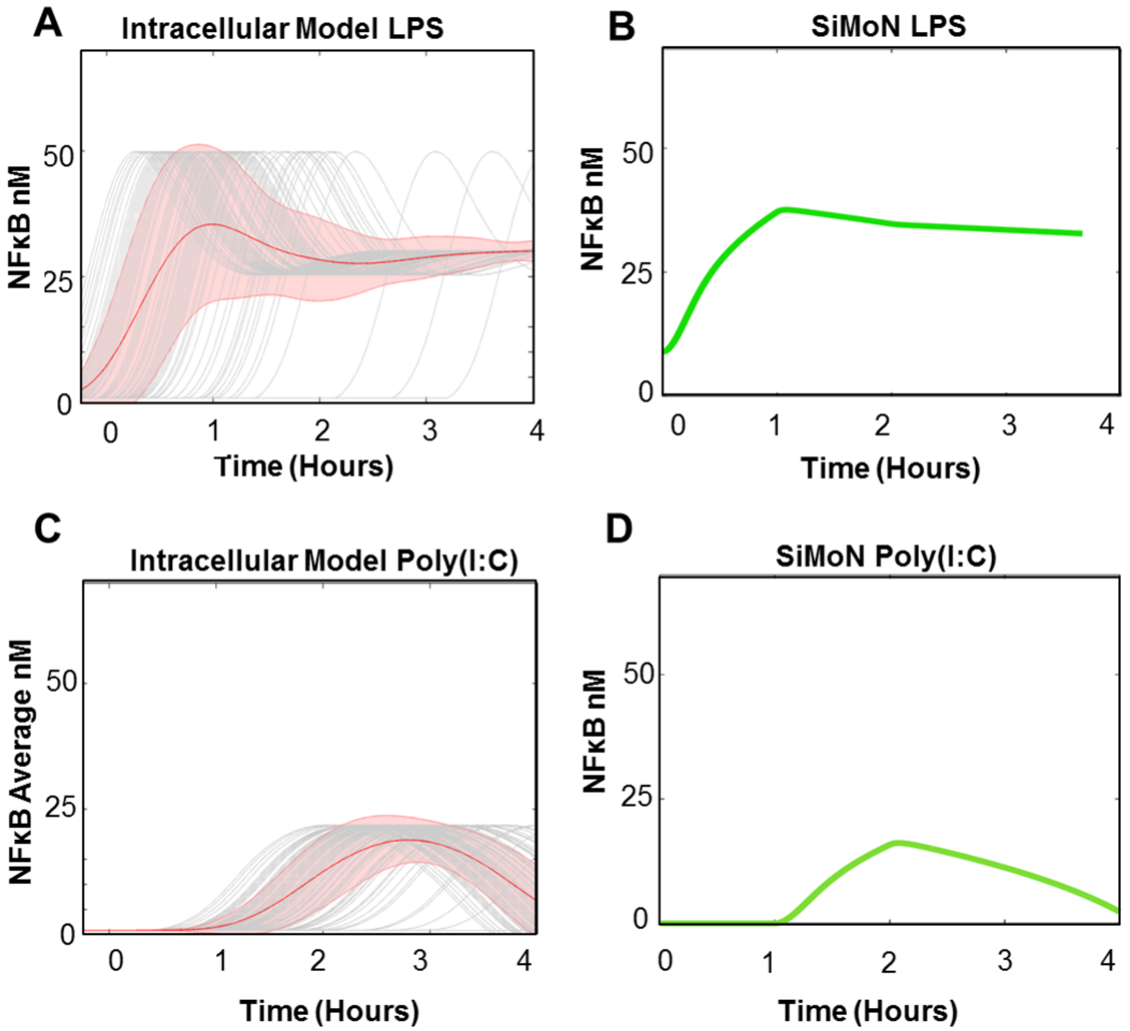

Supplementary Figure 2. **A)** 1000 single-cell time-course simulations with normally distributed inducible IκB transcription delay in response to simulated LPS input curve of IKK activity (light grey). Mean and standard deviation of 1000 simulations (red shaded region) (Werner et al, 2005). **B)** A single time-course simulation of the Simplified Model of NFκB (SiMoN) presented here in response to LPS input curve of IKK activity. **C)** 1000 single-cell time-course simulations with normally distributed inducible IκB transcription delay in response to simulated poly(I:C) input curve of IKK activity (light grey). Mean and standard deviation of 1000 simulations (red shaded region) (Werner et al, 2005). D) A single time-course simulation of SiMoN presented here in response to poly(I:C) input curve of IKK activity.

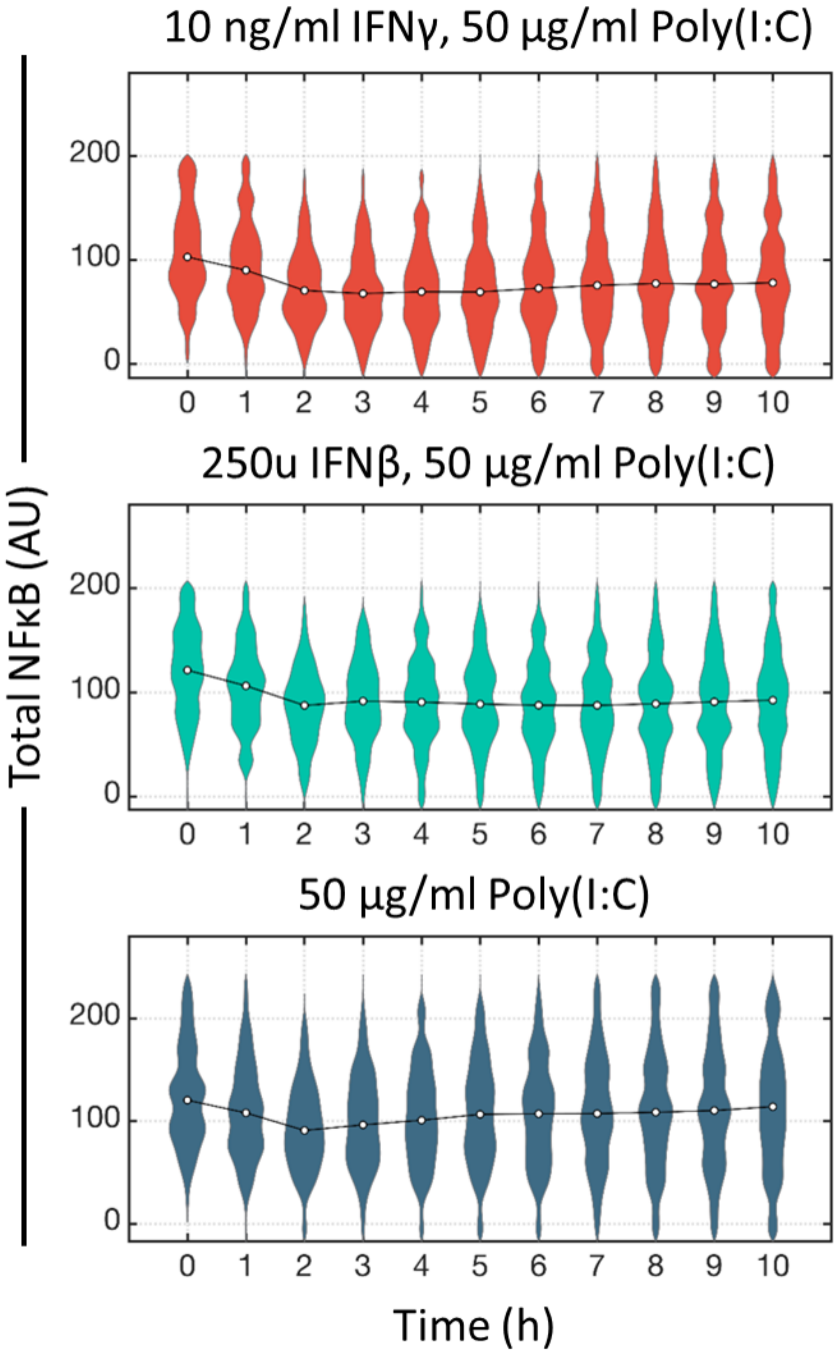

Supplementary Figure 3. Violin plots of total RelA-mVenus fluorescence in BMDMs (aribitrary units), stimulated with 50 µg/ml Poly(I:C) (bottom), co-stimulated with 250u IFNβ and 50 µg/ml Poly(I:C) (middle),10 ng/ml IFNγ and 50 µg/ml Poly(I:C) (top). Fluorescence of each cell is normalized by cytoplasmic area and the average (mean) within each 1 hour window is plotted.

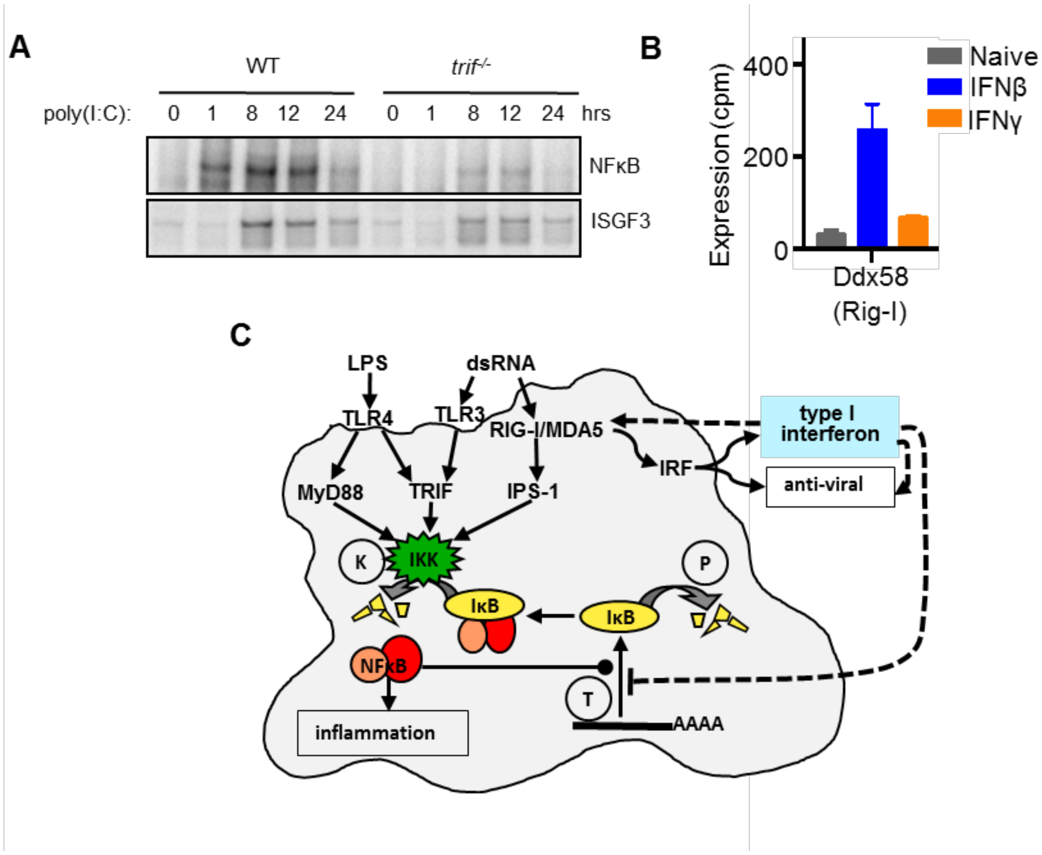

Supplementary Figure 4. A) EMSA for NFκB and ISGF3 in response to poly(I:C) in wild type (WT) and *trif*$^{-/-}$ BMDMs. **B)** Expression of Rig-I mRNA in human macrophages pre-stimulated with IFNβ and IFNγ, quantified from (Cheng et al, 2019). **C)** Schematic of the mechanism by with type I interferons amplify weak NFκB activating signals. Type I interferons inhibit IκBα translation and activate IKK-dependent IκBα degradation through RIG-I/IPS-1.

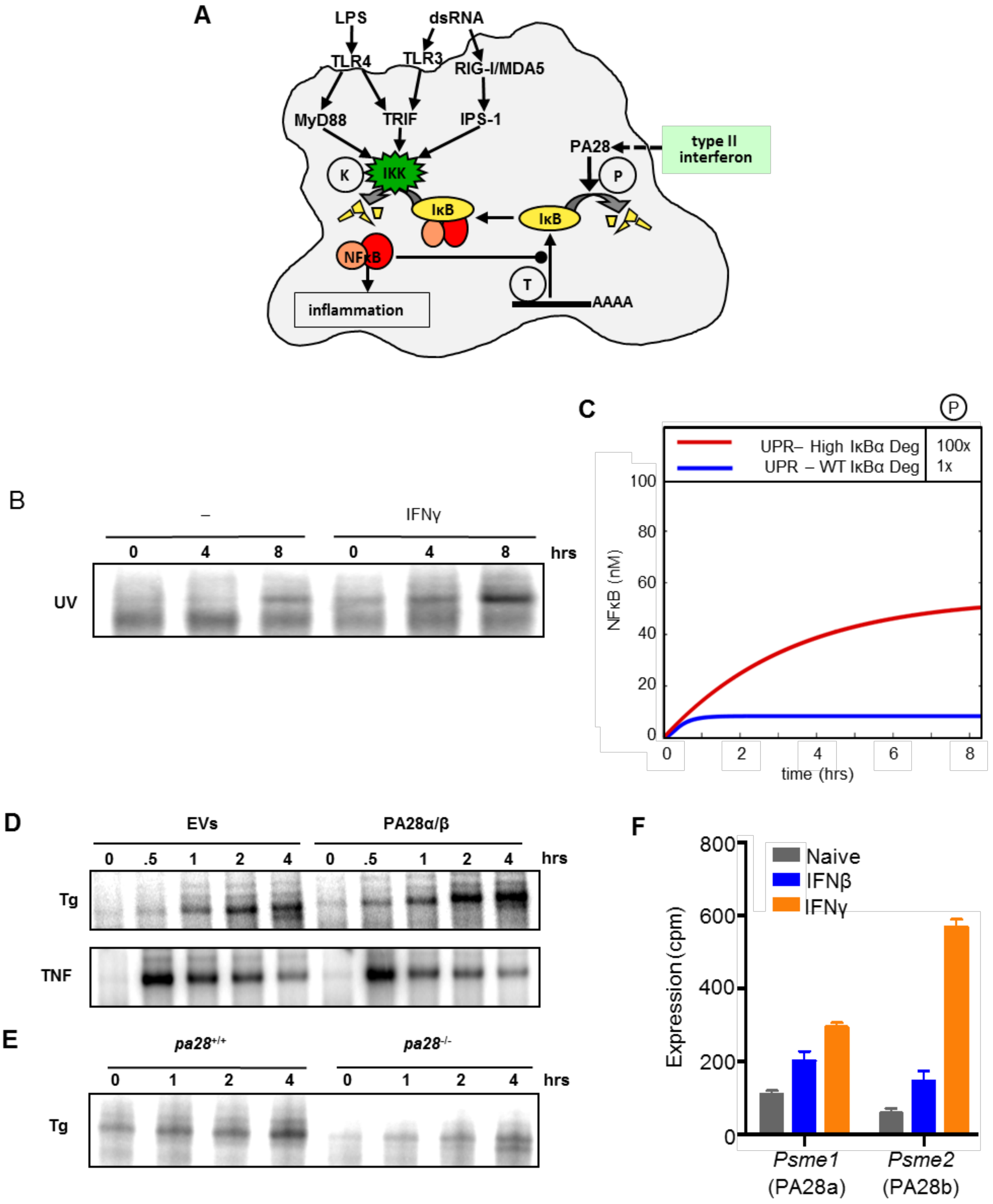

Supplementary Figure 5. A) Schematic of the mechanism by with type II interferon amplify weak NFκB activating signals. IFNγ induces free IκBα degradation in a PA28 proteasome dependent manner. **B)** NFκB EMSA in response to UV radiation in MEFs. **C)** Simplified model simulations with weak IKK activating input curve representing the unfolded protein response

(UPR). Default IκBα degradation rates (blue) and 100 fold increased IκBα degradation rates representative of pa28 overexpression experiments (red). **D)** NFκB EMSA in response to thapsigargin (Tg) and TNF in MEFs retrovirally transduced with PA28α/β over expression and empty vector (EV) controls. **E)** NFκB EMSA in response to thapsigargin (Tg) in wild-type (*pa28*$^{+/+}$) and pa28-deficient MEFs. **F)** Expression of PA28a and PA28b mRNA in human macrophages pre-stimulated with IFNβ and IFNγ, quantified from Cheng et al (2019).

References

Cheng Q, Behzadi F, Sen S, Ohta S, Spreafico R, Teles R, Modlin RL, Hoffmann A (2019) Sequential conditioning-stimulation reveals distinct gene-and stimulus-specific effects of Type I and II IFN on human macrophage functions. *Scientific reports* **9:** 5288

Le Novere N, Hucka M, Mi H, Moodie S, Schreiber F, Sorokin A, Demir E, Wegner K, Aladjem MI, Wimalaratne SM (2009) The systems biology graphical notation. *Nature biotechnology* **27:** 735-741

Werner SL, Barken D, Hoffmann A (2005) Stimulus specificity of gene expression programs determined by temporal control of IKK activity. *Science* **309:** 1857-1861
